# Editorial: Environmental racism: consideration of structural racism in environmental epidemiology

**DOI:** 10.3389/fepid.2024.1472215

**Published:** 2024-09-02

**Authors:** Jaime Madrigano, Genee S. Smith

**Affiliations:** Bloomberg School of Public Health, Johns Hopkins University, Baltimore, MD, United States

**Keywords:** environmental epidemiology, environmental justice, methods, environmental health disparities, structural racism

**Editorial on the Research Topic**
Environmental racism: consideration of structural racism in environmental epidemiology

Disparities in environmental hazards and amenities that fall along racial lines have been a well-described pattern in the U.S. for decades (1). Communities of color are more likely to be exposed to higher levels of pollution, are at greater risk for climate-related hazards, and benefit from fewer environmental amenities than other communities. Recent efforts to map upstream, structural determinants of discrimination and racism (e.g., historical redlining) have found associations with present-day distributions of environmental burdens and amenities, as well as health outcomes. Yet, despite recognition of structural racism as a crucial driver of environmental injustice, the measurement and analysis of the role of structural racism in environmental epidemiology has been limited. This Research Topic highlights the myriad of ways that structural racism may play a role in environmental epidemiology studies with consideration for study design.

Wien et al. raise important considerations for the rapidly evolving interest in incorporating structural racism into epidemiologic studies, generally. Although the recognition of the role of structural racism in health is an important and much-needed evolution in the field, its sudden uptake risks production of scholarship that is not grounded in relevant theory. Among a number of important recommendations, the authors reiterate calls from others (2, 3) and stress the importance of using historical and theoretical approaches that are shaped by theory and methods outside of epidemiology, applying this knowledge to evaluate what measures of structural racism are most appropriate for the research question, conceptualizing the population of interest not just by individual attributes but as shaped by intentional policy, critically evaluating the “baked-in” assumptions and limitations of secondary data sources, and partnering with experts across fields and from affected communities.

For environmental epidemiology, specifically, often concerned with the geographic characterization of environmental and climate hazards through measurement and modeling, the “baked-in” assumptions and limitations of secondary data used to assess environmental exposures are a critical consideration. Aune et al. compared three sources of exposure data for extreme precipitation events—weather stations in the Global Historical Climatology Network (GHCN) and gridded precipitation estimates from the Parameter-elevation Relationships on Independent Slopes Model (PRISM) and the North American Land Data Assimilation System (NLDAS). They found fair to moderate agreement among these three sources but important differences in level of agreement by race/ethnicity, socioeconomic status, and social vulnerability of the local area. The lower density of environmental monitors in certain parts of the country (e.g., rural areas) and the potential discriminatory siting of environmental monitors has been discussed previously (4). However, Aune et al. provide an important reminder of the inherent limitations of measured and modeled data in the potential for differential exposure misclassification across a study population. Due to the interconnectedness of structural racism, racial residential segregation, and the ability to accurately assess exposures through measured and modeled data, environmental epidemiologists should be considering the influence of structural racism, not only as an upstream determinant of health, but also within the context of their exposure assessment. Further, as Aune et al. recommend, affected communities should be engaged to determine their priorities for surveillance of environmental hazards to assure that future exposure assessment improvements adequately address community concerns.

An example of the type of community engagement that is needed to overcome exposure assessment challenges that may result from structural racism is the work of Aubourg et al. which highlights hyperlocal air monitoring coupled with authentic community engagement to document air pollution concentrations in an environmental justice community, where the nearest regulatory air monitor is over ten miles away. The study demonstrates how hyperlocal air monitoring can measure community-level air quality in the absence of regulatory monitors and be used to address resident concerns. The authors offer best practices for other community academic partnerships contending with similar issues of environmental justice, including centering the lived experiences of residents, intentionally prioritizing community needs and input, and explicitly acknowledging historical, systemic, and individual racism as drivers of environmental injustice. Aubourg et al. also recommend an emphasis on the multigenerational nature of the challenges environmental justice communities face, recognizing that racist policies and practices can have impacts across generations.

The multigenerational nature of structural racism is also emphasized in the article by Buxton et al., who make a compelling case for assessing multi-generational influences of pollutants and structurally racist policies and practices on health outcomes. The authors argue that this type of analysis is critical to understanding structural racism's long reach and enduring impact on health. For example, exposure to air pollution may have health impacts across generations that are not fully accounted for by only assessing exposure, or even prenatal exposure, for a given generation. The assessment of intergenerational air pollution may help to reconcile previously unexplained racial and ethnic health disparities in subsequent generations, and thus, may be useful for moving the environmental epidemiology literature toward a greater focus on modifiable policies and interventions, rather than the social construct of race.

When thinking about modifiable environmental amenities, such as green space, it is important to recognize that their health promotion potential may be mediated by discriminatory systems and structures that have impacted their spatial distribution and present-day access, as well as by personally mediated racism, which may affect subjective experience. In their study assessing how everyday discrimination affects experiences with nature, Schinasi and Lawrence remind us that racism manifests at multiple levels—structural, institutional, personally mediated, and internalized—and that these levels are interrelated. The authors found that individuals who reported a higher frequency of everyday discrimination were less satisfied with their experiences in nature and highlight the need to consider the person-, cultural-, and area-level contexts in which green spaces are situated when proposing green interventions. Understanding the historical legacy of structural racism, and its impact on access to environmental amenities, is critical, and extending these considerations beyond green space to other environmental interventions is warranted.

Collectively, the articles in this Research Topic highlight several important considerations for thinking about structural racism within the framework of environmental epidemiology. These papers help to move the field forward by reiterating previous calls (5) to focus on systems and policies, rather than ascribing racial differences to biological differences; develop a deeper understanding of the role of racism in environmental health disparities through the lens of historical context; and consider how structural racism affects the measurement and suitable time-window of environmental exposures in the study design. Future environmental epidemiology studies should explicitly describe the scientific rationale for including race/ethnicity as an exposure, confounder, or effect modifier and consider whether inclusion of race is appropriate or whether there is a measure of racism that may be better suited for the intended use. Further, environmental epidemiologists can work in interdisciplinary teams to develop new measures of racism that are particularly relevant for environmental health. The specific application of these concepts to environmental epidemiology is a welcome step forward for the field.

## References

[1] Bullard RD Mohai P Saha R Wright B. Toxic wastes and race at twenty: why race still matters after all of these years. Environmental Law. (2008) 38(2):371–411.

[2] Adkins-Jackson PB Chantarat T Bailey ZD Ponce NA. Measuring structural racism: a guide for epidemiologists and other health researchers. Am J Epidemiol. (2021) 191(4):539–47. 10.1093/aje/kwab239 PMC9077112 34564723

[3] Hardeman RR Homan PA Chantarat T Davis BA Brown TH. Improving the measurement of structural racism to achieve antiracist health policy. Health Aff (Millwood. (2022) 41(2):179–86. 10.1377/hlthaff.2021.01489 35130062 PMC9680533

[4] Grainger C Schreiber A. Discrimination in ambient air pollution monitoring? AEA Papers Proc. (2019) 109:277–82. 10.1257/pandp.20191063

[5] Payne-Sturges DC Gee GC Cory-Slechta DA. Confronting racism in environmental health sciences: moving the science forward for eliminating racial inequities. Environ Health Perspect. (2021) 129(5):55002. 10.1289/EHP8186 33945300 PMC8096378

